# Pulmonary Pneumocytoma: A Benign Rarity Behind a Suspicious Nodule

**DOI:** 10.7759/cureus.102132

**Published:** 2026-01-23

**Authors:** Krin Suhr, Florencia Belen Basile, Adolfo Rosales, Moises Rosenberg, Emiliano Navarro

**Affiliations:** 1 Thoracic Surgery, Instituto Alexander Fleming, Buenos Aires, ARG

**Keywords:** lung pathology, lung tumor, pulmonary pneumocytoma, sclerosing hemangioma, tumor

## Abstract

Pulmonary pneumocytoma is a rare benign tumor most often detected incidentally as a solitary pulmonary nodule. Although its radiological appearance is nonspecific, its histological complexity can mimic several malignant neoplasms, leading to potential overtreatment. We report a case of a 40-year-old female with a suspicious pulmonary lesion discovered during the evaluation of an unrelated oropharyngeal carcinoma. Surgical resection and histopathological analysis confirmed the diagnosis of pneumocytoma. This case highlights the importance of considering pneumocytoma in the differential diagnosis of solitary pulmonary nodules, particularly in middle-aged female patients. Early multidisciplinary evaluation is essential to avoid unnecessary radical treatment.

## Introduction

Pulmonary pneumocytoma is a rare benign lung tumor, first described by Liebow and Hubbell in 1956 as “sclerosing hemangioma” due to its prominent hemorrhagic and fibrotic features [1]. Although it was initially believed to be of vascular origin, subsequent studies demonstrated its epithelial nature, leading to its current designation as pneumocytoma [2-4]. This tumor accounts for less than 1% of all primary pulmonary neoplasms and occurs predominantly in middle-aged females [5].

In most cases, pulmonary pneumocytoma is detected incidentally during imaging studies performed for unrelated reasons. On computed tomography (CT), it usually appears as a well-circumscribed solitary pulmonary nodule, defined as a rounded lung opacity measuring up to 3 cm in diameter and surrounded by normal lung parenchyma, without associated atelectasis or lymphadenopathy [3,5]. These radiologic findings are nonspecific and overlap with those of malignant lesions such as lung adenocarcinoma and carcinoid tumors, as well as with benign or borderline entities, including papillary adenoma and other rare epithelial tumors [6]. Consequently, establishing an accurate preoperative diagnosis is often challenging, and many patients undergo surgical resection under the presumptive diagnosis of lung cancer [6].

Preoperative tissue diagnosis may be attempted; however, percutaneous or bronchoscopic biopsy is frequently limited in small or peripheral nodules and may yield insufficient or non-representative samples. Moreover, the cytological and architectural features of pneumocytoma can overlap with those of malignant epithelial tumors, potentially leading to diagnostic uncertainty. For these reasons, surgical resection is often pursued for both diagnostic and therapeutic purposes [6].

Histopathological examination supported by immunohistochemistry plays an important role in confirming the diagnosis. Nevertheless, immunohistochemical markers commonly expressed in pneumocytoma, such as thyroid transcription factor-1 (TTF-1), are not entirely specific, as they may also be positive in lung adenocarcinoma and papillary adenoma [6]. In contrast, neuroendocrine markers such as chromogranin A, synaptophysin, and CD56 are typically expressed in carcinoid tumors but are absent in pneumocytoma, aiding in the differential diagnosis. Despite its diagnostic value, immunohistochemistry may not be readily available in routine clinical practice due to cost and limited accessibility in some settings, further complicating the diagnostic process.

Although the clinical course of pulmonary pneumocytoma is benign and complete surgical excision is considered curative, its rarity and nonspecific clinical, radiologic, and immunohistochemical features make it a relevant differential diagnosis of solitary pulmonary nodules. Awareness of this entity among clinicians, radiologists, and pathologists is essential to avoid unnecessary extensive resections and to ensure appropriate patient management [7].

## Case presentation

A 40-year-old female patient with a medical history of endometriosis and pulmonary hypertension, a lifelong non-smoker, presented with progressive dysphagia and odynophagia. She denied respiratory symptoms such as cough, dyspnea, chest pain, or hemoptysis, and had no prior history of pulmonary disease. Baseline laboratory tests were within normal limits, and no previous chest imaging studies were available for comparison.

**Figure 1:**
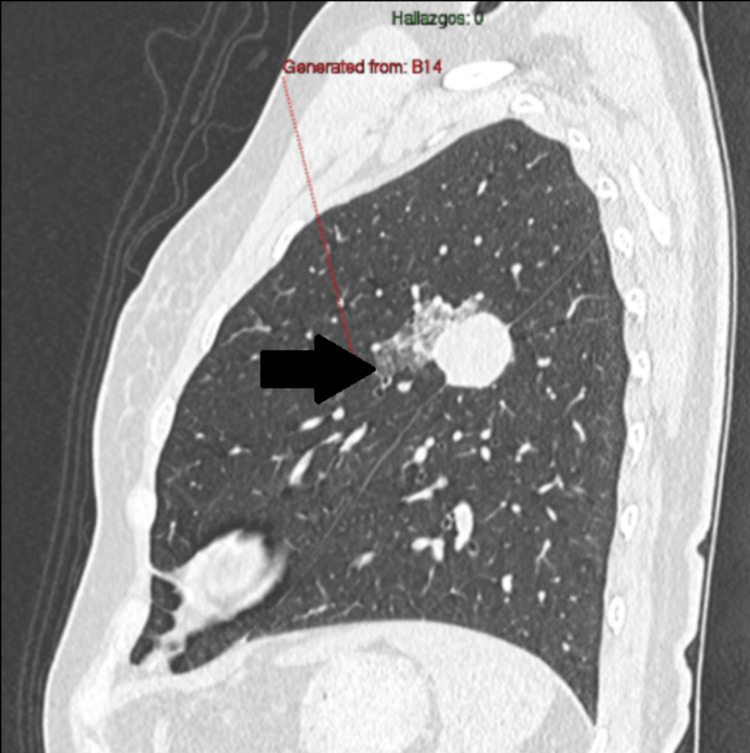
Ground-glass halo surrounding the interlobar (intercisural) nodule.

**Figure 2:**
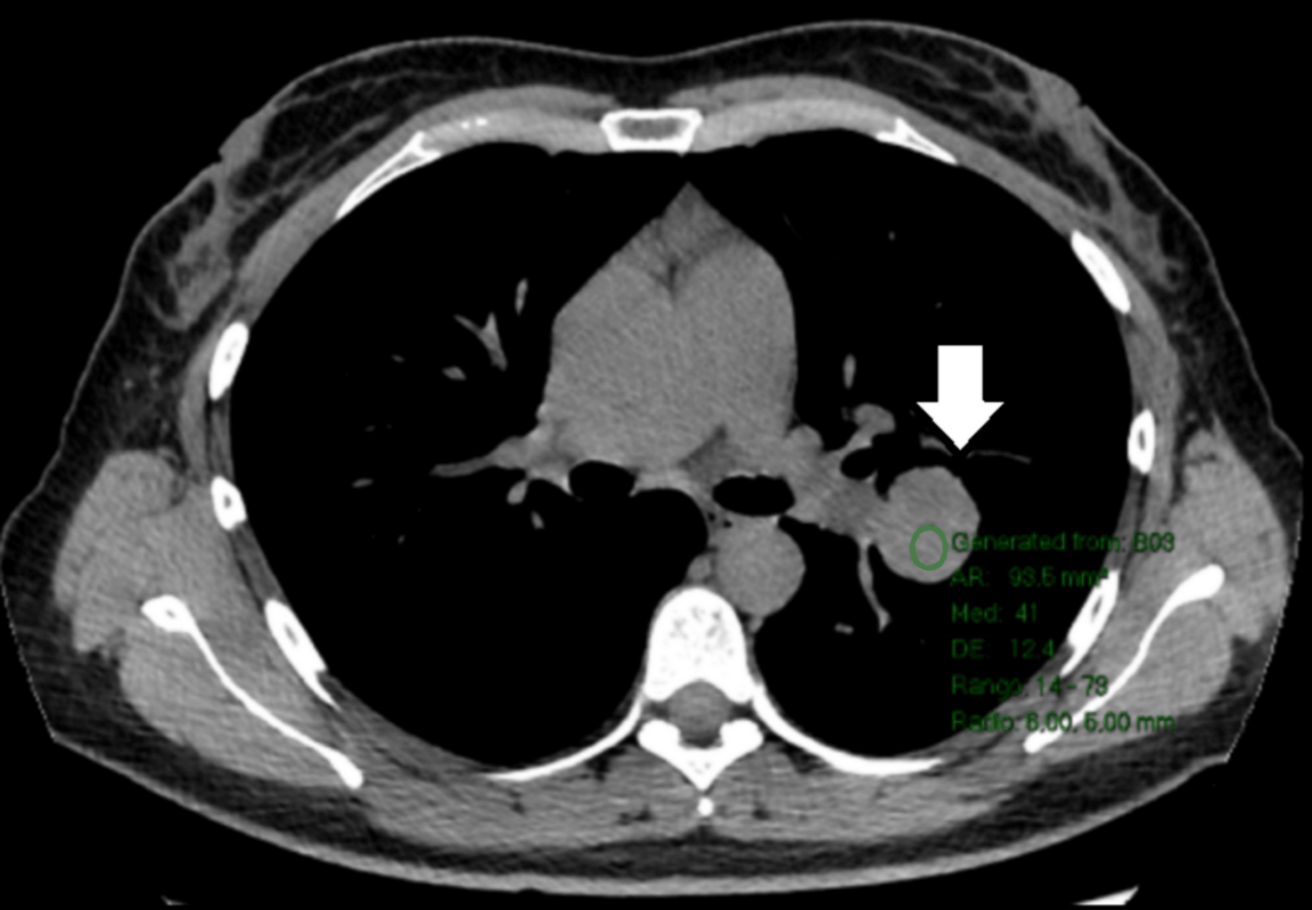
Intermediate density without intravenous contrast.

**Figure 3:**
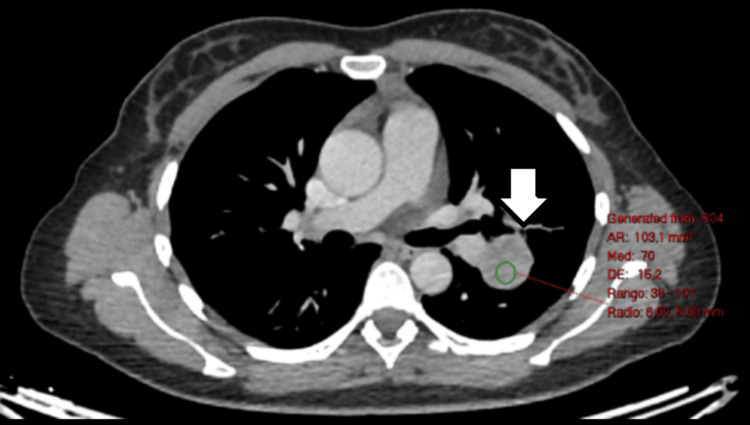
Moderate enhancement post intravenous contrast.

**Figure 4:**
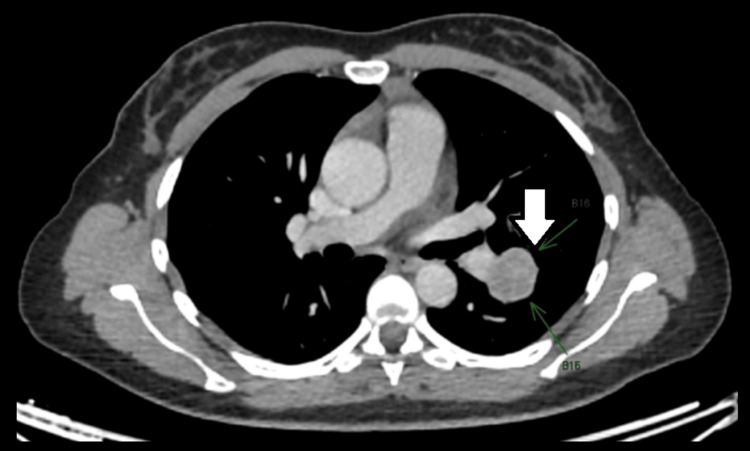
Nodular pseudocapsule.

Nasofibrolaryngoscopy revealed asymmetry at the base of the right side of the tongue and a small lesion involving the vallecula and the right glossopharyngeal fold. A computed tomography (CT) scan of the neck demonstrated irregular mucosal thickening obliterating the vallecular space, with subtle homogeneous enhancement. As an incidental finding, a round, well-defined soft tissue-density pulmonary mass was identified at the junction of the upper and middle thirds of the left oblique fissure, measuring 32 mm in diameter, without enhancement after intravenous contrast administration (Figures 1-4).

**Figure 5:**
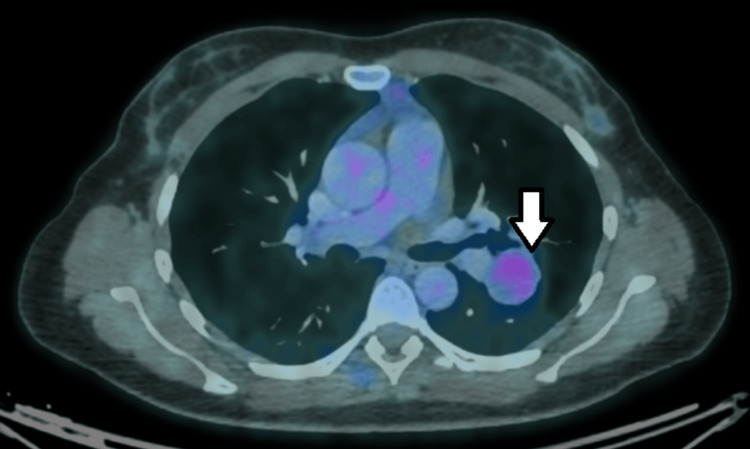
Mild uptake in PET/FDG (SUV = 2.4) of the pulmonary nodule. PET: positron emission tomography; FDG: fluorodeoxyglucose; SUV: standardized uptake value.

A positron emission tomography-computed tomography (PET-CT) scan showed a left pulmonary lesion with nonspecific morphological and metabolic characteristics, measuring 32 × 28 mm, with a standardized uptake value (SUV) of 2.4. Additionally, focal tracer uptake was observed in the right pharyngeal tonsil, which appeared enlarged, with a maximum SUV of 2.6 (Figure 5).

A biopsy of the right tonsil revealed in situ squamous cell carcinoma, and the lesion was subsequently treated by endo-oral excision.

Given the coexistence of a pulmonary nodule and a head and neck squamous cell carcinoma, a diagnostic dilemma arose regarding the possibility of a synchronous primary lung tumor versus pulmonary metastasis. The case was therefore discussed at a multidisciplinary tumor board, as is routine practice in our institution for complex oncological cases. In view of the indeterminate imaging findings and the potential oncologic implications, surgical excision of the pulmonary lesion with mediastinal lymph node dissection was recommended.

**Figure 6:**
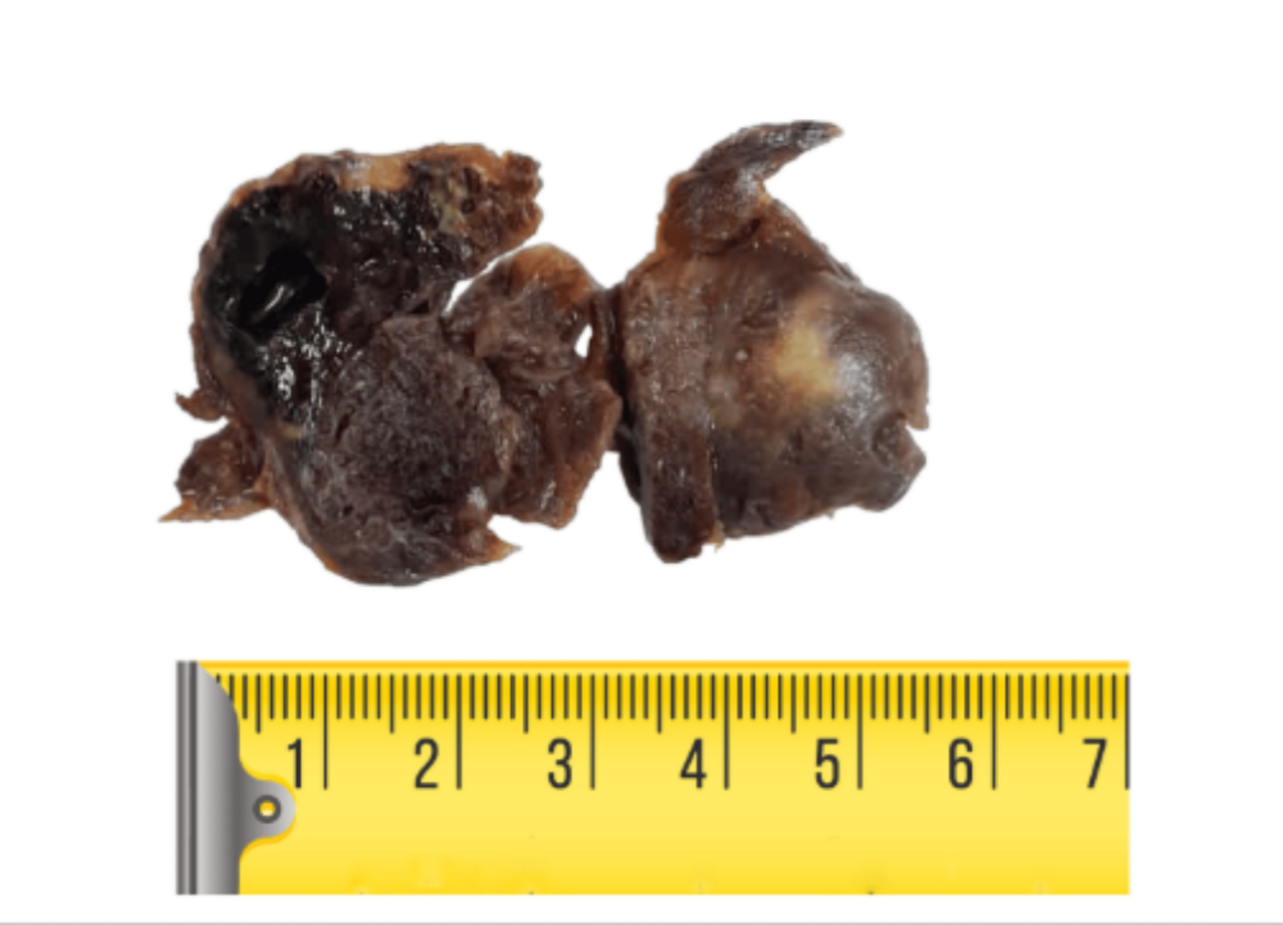
Surgical specimen of interlobar (intercisural) pulmonary nodule.

**Figure 7:**
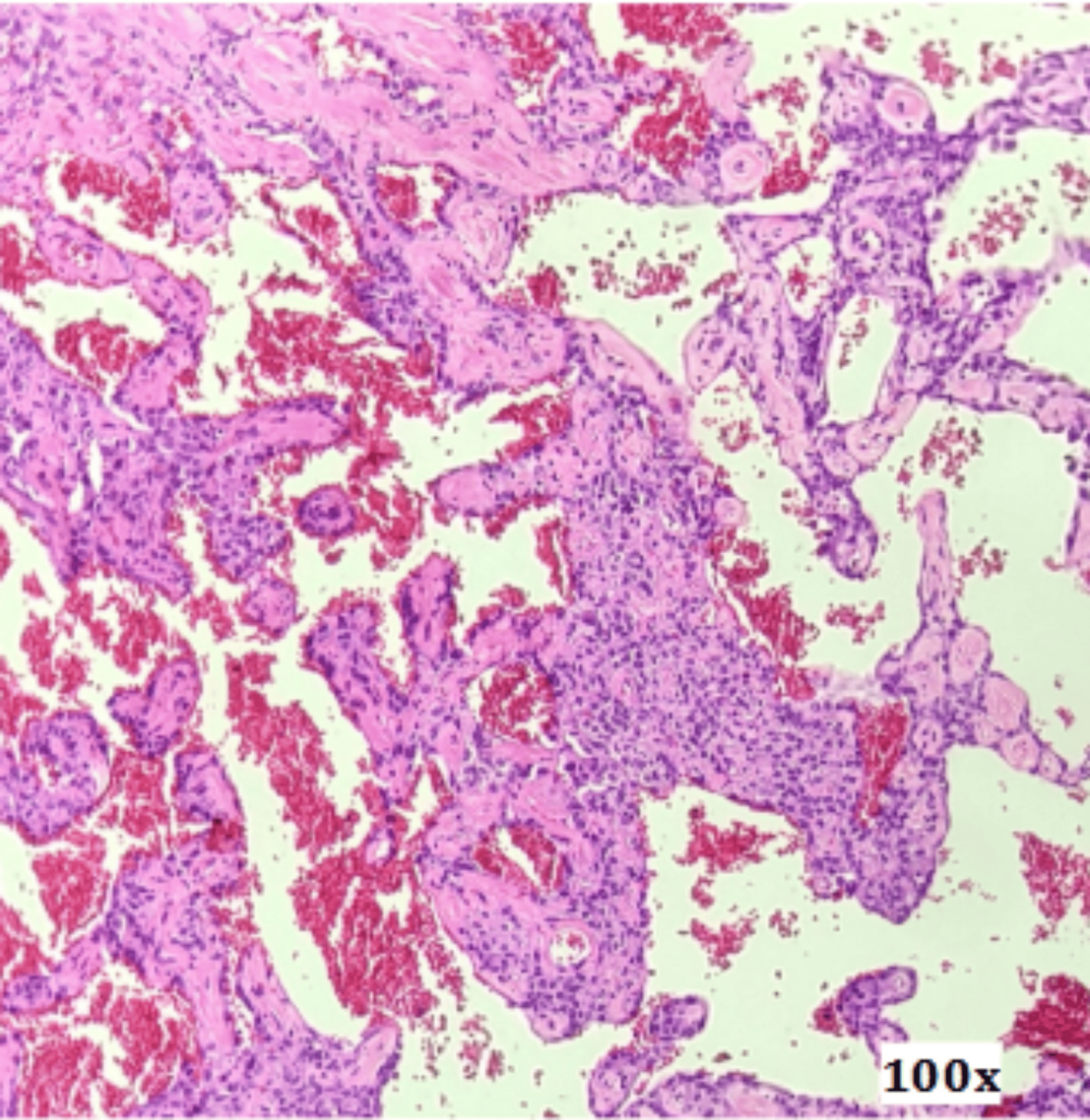
Surgical specimen microscopy, 100x magnification.

**Figure 8:**
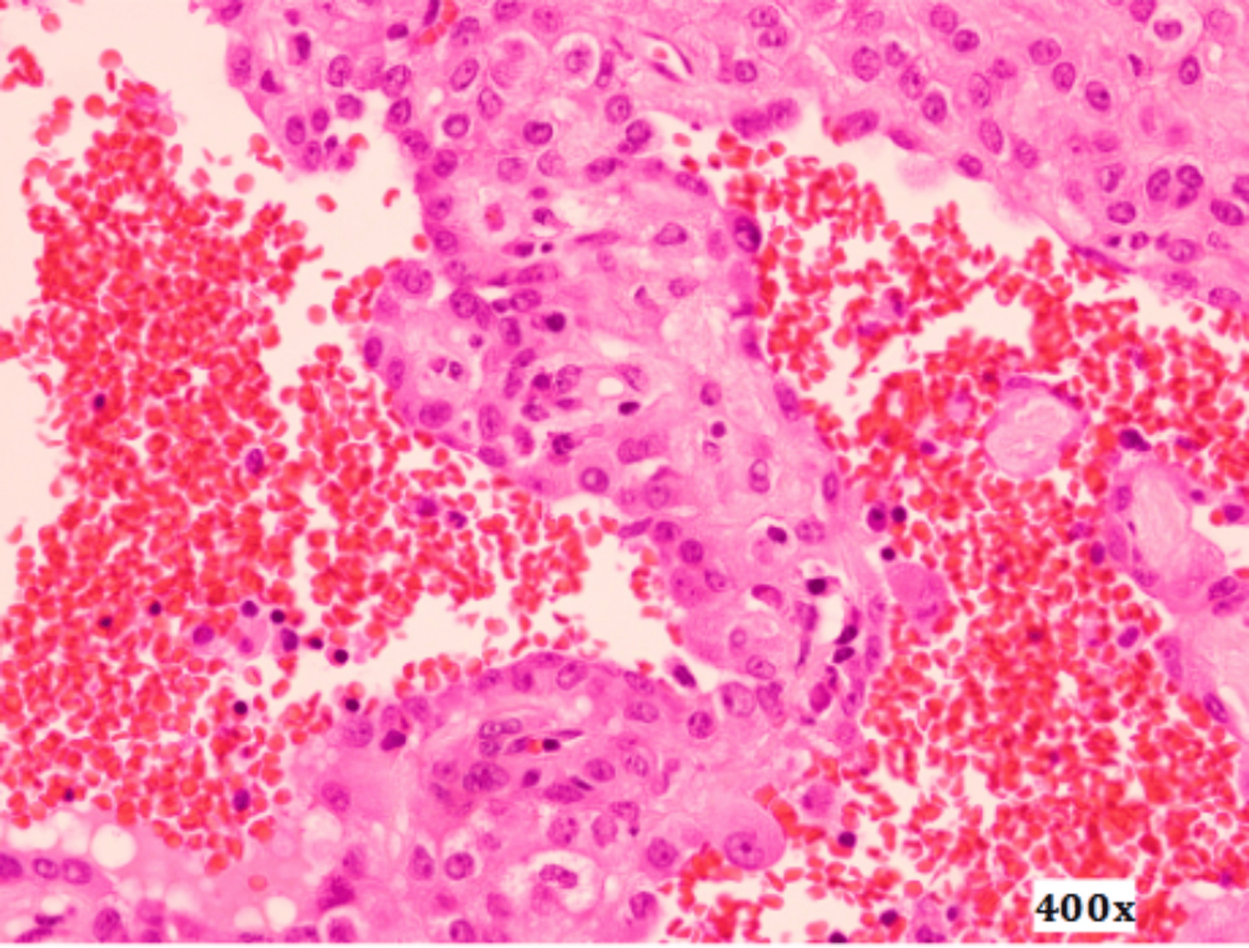
Surgical specimen microscopy, 400x magnification. Proliferation is composed of papillary structures lined by cells with round, prominent nuclei, interspersed with areas of lightly spindle-shaped cells and regions of hemorrhage.

Histopathological examination of the resected specimen revealed lung parenchyma containing a well-circumscribed nodular lesion composed of a cellular proliferation with papillary structures featuring thick fibrovascular cores, lined by cells with round, prominent nuclei. These areas alternated with solid nests of slightly spindle-shaped cells. Extensive hemorrhagic areas and clusters of histiocytes were also identified (Figures 6-8).

Immunohistochemical analysis demonstrated positivity for epithelial membrane antigen (EMA), cytokeratin 7 (CK7), TTF-1, cytokeratin AE1/AE3, and CD68, while staining for CD34, CD31, ERG, and CK8/18 was negative. These findings helped exclude alternative diagnoses such as adenocarcinoma, carcinoid tumor, and vascular neoplasms.

Based on the histopathological and immunophenotypic features, a diagnosis of pulmonary pneumocytoma was established. Lymph nodes from stations 5 and 10 showed only reactive adenitis, with no evidence of metastatic disease.

## Discussion

Pulmonary pneumocytoma is a rare benign pulmonary neoplasm with a complex histological architecture. Advances in immunohistochemistry and electron microscopy have established its origin in primitive respiratory epithelium, specifically type II pneumocytes, supported by the expression of EMA, pancytokeratin, TTF-1, and napsin A [3,4,6,8]. The two distinct cellular populations observed in this tumor are considered different maturational stages within a uniform monoclonal process [9]. Four histological patterns have been described, including papillary, solid, sclerotic, and hemorrhagic, which frequently coexist within the same lesion [3].

Owing to this histological complexity, the differential diagnosis is broad and includes bronchioloalveolar adenocarcinoma, carcinoid tumor, papillary adenoma, angiosarcoma, and mesothelioma [3,6]. Several of these entities represent aggressive malignancies requiring radical oncological treatment, underscoring the importance of an accurate diagnosis. In particular, adenocarcinoma and papillary adenoma may share epithelial marker expression, while carcinoid tumors may present overlapping architectural features; however, the absence of neuroendocrine marker expression and the presence of two distinct cell populations favor the diagnosis of pneumocytoma.

Despite occasional reports of lymph node involvement, pulmonary pneumocytoma is still considered a benign neoplasm, based on its characteristic histological features, including minimal cytologic atypia and absence of mitotic activity, and the lack of demonstrated impact of lymphatic involvement on prognosis [10]. Surgical resection is therefore regarded as curative.

Intraoperative pathological assessment may pose a significant challenge, as pneumocytoma can be misinterpreted as a low-grade malignant tumor. Although frozen section analysis can be helpful in selected cases, it was not performed in the present case due to the central location of the lesion and the associated risk of vascular injury, given the hemorrhagic nature of the tumor. For similar reasons, preoperative percutaneous biopsy was avoided, as needle sampling may carry a risk of vascular damage and bleeding and may also provide limited or non-representative tissue, potentially leading to diagnostic uncertainty [11].

Clinically, pneumocytoma most often presents as an asymptomatic solitary pulmonary nodule, with no specific or characteristic symptoms. It predominantly affects middle-aged women, with a reported female-to-male ratio of approximately 5:1 [3,6,12]. Tumor size varies widely, reaching up to 70 mm in some reports, although the majority of lesions measure less than 30 mm in diameter [12]. When symptoms occur, they are nonspecific, with chest pain being the most frequently reported complaint [13].

Radiologically, pneumocytoma typically appears as a well-circumscribed peripheral pulmonary lesion [4]. Rare cases of endobronchial growth or multifocal and bilateral disease have been described [14-17]. Both CT and PET-CT findings are nonspecific, and pneumocytoma may exhibit low to moderate fluorodeoxyglucose uptake, overlapping with other low-grade malignant or metastatic tumors [18,19]. Consequently, PET-CT has a limited role in distinguishing pneumocytoma from other pulmonary neoplasms with low metabolic activity.

The present case is particularly notable due to the coexistence of a pulmonary nodule and a synchronous head and neck squamous cell carcinoma, which created a significant diagnostic dilemma regarding the possibility of pulmonary metastasis versus a second primary lung tumor. This uncommon clinical scenario highlights the importance of considering pneumocytoma in the differential diagnosis of pulmonary nodules, even in oncological patients, to avoid overtreatment and unnecessary extensive resections.

## Conclusions

Pulmonary pneumocytoma is an extremely rare benign neoplasm that typically presents as a solitary, well-circumscribed pulmonary nodule with nonspecific radiological features. It should be considered in the differential diagnosis, particularly in middle-aged female patients presenting with a well-defined pulmonary lesion, and should not be excluded even in patients with other primary malignancies.

Surgical excision is both diagnostic and therapeutic; however, accurate preoperative assessment is essential to avoid overtreatment. A multidisciplinary approach, involving thoracic surgeons, radiologists, pathologists, and oncologists, is crucial for appropriate decision-making and optimal patient management.
